# Electrode Arrays for Detecting and Modulating Deep Brain Neural Information in Primates: A Review

**DOI:** 10.34133/cbsystems.0249

**Published:** 2025-05-02

**Authors:** Siyu Zhang, Yilin Song, Shiya Lv, Luyi Jing, Mingchuan Wang, Yu Liu, Wei Xu, Peiyao Jiao, Suyi Zhang, Mixia Wang, Juntao Liu, Yirong Wu, Xinxia Cai

**Affiliations:** ^1^ State Key Laboratory of Transducer Technology, Aerospace Information Research Institute. Chinese Academy of Sciences, Beijing 100190, China.; ^2^ School of Electronic, Electrical and Communication Engineering, University of Chinese Academy of Sciences, Beijing 100049, China.

## Abstract

Primates possess a more developed central nervous system and a higher level of intelligence than rodents. Detecting and modulating deep brain activity in primates enhances our understanding of neural mechanisms, facilitates the study of major brain diseases, enables brain–computer interactions, and supports advancements in artificial intelligence. Traditional imaging methods such as magnetic resonance imaging, positron emission computed tomography, and scalp electroencephalogram are limited in spatial resolution. They cannot accurately capture deep brain signals from individual neurons. With the progress of microelectromechanical systems and other micromachining technologies, single-neuron level detection and stimulation technology in rodents based on microelectrodes has made important progress. However, compared with rodents, human and nonhuman primates have larger brain volume that needs deeper implantation depth, and the test object has higher safety and device preparation requirements. Therefore, high-resolution devices suitable for long-term detection in the brains of primates are urgently needed. This paper reviewed electrode array devices used for electrophysiological and electrochemical detections in primates’ deep brains. The research progress of neural recording and stimulation technologies was introduced from the perspective of electrode type and device structures, and their potential value in neuroscience research and clinical disease treatments was discussed. Finally, it is speculated that future electrodes will have a lot of room for development in terms of flexibility, high resolution, deep brain, and high throughput. The improvements in electrode forms and preparation process will expand our understanding of deep brain neural activities, and bring new opportunities and challenges for the further development of neuroscience.

## Introduction

The brain is the most complex and mysterious biological information system and the ultimate territory of scientific exploration. Its vast neural network, precise signal transmission, and complex structure constitute the basis of human cognition, emotion, and behavior. Neural information transmission includes the discharge of neurons and neurotransmitter release. Detecting neuronal firing provides insights into how neurons direct behavior, aiding in the study of sensory perception, motor actions, and cognition [1]. Assessing neurotransmitter release is essential for understanding synaptic transmission and its role in neurological conditions. For instance, excessive glutamate release can lead to excitotoxicity, contributing to diseases such as multiple sclerosis, amyotrophic lateral sclerosis, and Parkinson’s disease [2]. It is of great significance to detect these 2 kinds of information. Microelectrode technology has been developed in different forms for a long time, and important progress has been made in the neural information detection of rodents, nonhuman primates (NHPs), and human brains. Among them, primates have a more developed central nervous system and a higher level of intelligence than rodents. Detecting neural information in primates is the neural underpinnings for studying complex cognitive functions, such as decision-making, social interactions, and problem-solving, which are more analogous to human processes than those in rodents. While deep brain and whole-brain recordings are possible in rodents, primates possess specialized neural circuits that are crucial for studying human-like functions. For example, by recording electrophysiological activity from both superficial and deep brain structures in primates, capturing neural activity related to advanced visual processing, decision-making, and motor control, functional connections between cells in the cortex and deep brain of primates can be studied, as well as brain-wide neural recordings [3]. The detection and modulation of neural information in primate brains are important for diagnosing and treating major brain diseases, understanding neural mechanisms, and achieving brain–computer interaction.

**Fig. 1.**
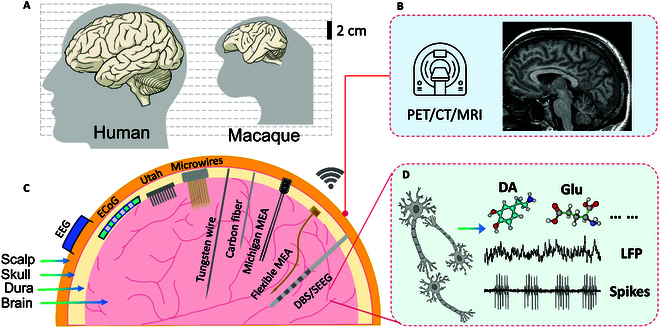
Schematic diagram of brain neural information detection technologies. (A) Approximate brain size of human and macaque, which is a widely used experimental nonhuman primate. (B) Imaging methods for the detections of brain structure and function. (C) Different types of neural electrode devices for human and nonhuman primates. (D) Dual-mode information derived from neurons, including electrophysiological signals [spikes and local field potential (LFP)] and neurotransmitters [dopamine (DA), glutamate (Glu), etc.].

However, because primates’ brain volume and size (Fig. 1A) are hundreds to thousands of times larger than rodents, the structure is more complex, and the relevant experiments need strict ethical review, the development of deep brain neural detection and stimulation technology in primates is less mature than that of rodent devices. There are still many difficulties and needs in high-resolution, high-throughput, deep brain detection, which has a lot of room for development. Therefore, this paper focuses on the electrode device technologies used for detecting and stimulating deep brain neural information in primates and makes an in-depth summary and analysis from the perspective of electrode type, device structure, application, and improvement.

The detection and modulation of brain neural information play a vital role in neuroscience. The existing traditional imaging methods, such as magnetic resonance imaging and positron emission computed tomography (Fig. 1B), can examine brain structure and metabolic functions noninvasively, but they cannot directly reflect information related to neural encoding in the brain. Different types of neural electrode devices have long been used for electrophysiological recordings from the scalp to the deep brain (Fig. 1C). Although electrodes for scalp electroencephalogram (EEG) electrodes and cranial endothelial electrocorticogram (ECoG) recordings are atraumatic to brain tissue and are applied in NHPs and clinical patients [4], their spatial resolution is limited. They cannot directly contact the neurons and usually have much greater size than neurons, so the accurate neural information from single neurons in deep brain were missed, and only macroscopic field potentials could be recorded. The Utah electrode array fabricated by microelectromechanical systems (MEMS) technology can detect multichannel single-cell action potentials, but due to its fabrication process, the electrode length is around 1 to 2 mm, and the detection depth stays in the cortex. Compared to rodents, primates possess larger brain volume and require greater implantation depth. For instance, the brain volume of rhesus monkey is 79.1 ± 3.4 cm^3^, and the brain volume of humans is 1,298.9 ± 52.0 cm^3^ [5]. According to the brain atlas, the distance from the vertex of the skull to the base of the brain in the rhesus monkey is approximately 50 mm. The subthalamic nucleus (STN) in macaques, which serves as a critical target for deep brain stimulation (DBS) surgery, is located at a depth ranging from approximately 32 to 36 mm [5,6]. Detecting deep brain nuclei requires electrodes with greater length to reach the target regions and higher mechanical strength to penetrate dense tissue without bending or breaking while maintaining high throughput and spatiotemporal resolution for precise neural recordings. In addition, research in primates involves higher safety standards, which puts forward higher requirements for electrode preparation and experimental design. Therefore, researchers are committed to developing new electrode array devices for primates to improve the recording effect and reliability.

The detection of deep brain neural activity in NHPs plays a crucial role in the diagnosis and treatment of brain diseases, the development of brain–computer interface (BCI) technology, and the study of neural information processing and cognitive mechanisms. Given the close anatomical and functional similarities between NHP and human brains, invasive electrophysiological recordings in NHPs provide valuable insights into the neural mechanisms underlying cognition, motor control, and neurological disorders. This approach is particularly important for the diagnosis and treatment of brain diseases, such as Parkinson’s disease, Alzheimer’s disease, and depression, where DBS has emerged as an effective therapeutic strategy [7]. Moreover, high-precision neural signal acquisition is essential for the development of BCI technology, especially in application fields such as motion control and neurological rehabilitation [8]. Additionally, studying deep brain neural information in NHPs contributes to a deeper understanding of fundamental cognitive processes, including decision-making, memory, and spatial navigation. By improving electrode techniques and implantation strategies, researchers can enhance the long-term stability and signal quality of deep brain electrodes, facilitating both clinical applications and fundamental neuroscience research.

The deep brain contains a lot of direct neural information from different brain regions, which is closely related to many neurological diseases, such as the thalamus and hippocampus related to epilepsy, the basal ganglia and striatum related to Parkinson’s disease, and the hippocampus and entorhinal cortex related to spatial cognitive impairment. The electrode devices obtain electrophysiological signals, including neural spikes and local field potentials (LFPs), and electrochemical signals (Fig. 1D) in the deep brain, which can be used to study brain mechanisms and brain disease. Therefore, researchers actively explore and develop new deep brain electrodes and related technologies for multi-mode neural detection and stimulation to expand our research on the brain circuit mechanisms and the treatment methods for brain neurological diseases. In the past, most articles summarized electrodes for rodents or general purpose, and few reviews focused on the deep brain of primates. In the future, BCI technology will have an increasing demand for primate deep brain electrodes. Therefore, it is urgent to review and analyze this technology in depth, which can lay a foundation for the further development of electrode devices in the future.

With the progress of technology, researchers have made a series of important breakthroughs in the detection of single-neuron discharges in the deep brain of primates, and even made noteworthy progress in the detection of neurotransmitters, providing basic means and tools for studying the neural information of primates. The development of these technologies has provided new possibilities for us to explore the function of neural circuits and the interaction of brain regions. These technologies include but are not limited to microelectrode devices based on MEMS technology, optogenetics, and others. The devices can be implanted into the deep brain and record the neuronal signals with high spatial-temporal resolution, which include single-site electrodes or multi-site electrodes [9]. Multi-site microelectrode array (MEA) devices are usually fabricated by MEMS technology on different substrates such as metal, silicon, ceramic, or polymer. When designing and preparing electrodes, especially for primates, many factors need to be comprehensively considered, such as electrode size, biocompatibility of materials, electrode impedance, and electrode stability. The material [10,11], tip shape [12,13], and insertion speed [14,15] of MEAs are related to the stress generated when implanted into the deep brain, which in turn affects brain tissue. Through continuous improvement and optimization of the design and fabrication processes, the current electrode device has high performance and reliability, and can stably record neural signals in long-term experiments.

## Development History of Deep Brain Electrode Array Devices

Horsley and Clarke [16] developed a brain stereotactic device in 1908 to locate brain regions and guide electrodes. Subsequently, Aubrey Mussen designed the first stereotaxic device for the human brain in 1918 [17]. In 1947, Hayne et al. [18] first used the Horsley–Clarke stereotactic instrument to study the subcortical neurophysiological activities of epileptic patients. They guided the multi-electrode needle to the correct position in human deep brain structures to record the seizure discharges and perform surgery. The multi-electrode needle is composed of 8 silver rings 2 mm in width and separated by 2 mm of insulating material, which can record LFPs in different brain regions. With the development of computers and the progress of technology, the software tool developed by Miocinovic et al. [19] can 3-dimensionally (3D) visualize the data of images and neurophysiology related to deep brain of NHPs, predict the anatomical location of the needle, and evaluate its accuracy. Due to the primate brain’s large size, the presence of many internal nuclei, a broad diversity of neurons, the large depth required for electrode implantation, and the challenge of precise implanting location by the surgical planning system, the invention of these stereotaxic instruments and software has made contributions to the precise positioning of DBS electrodes and better application of electrodes.

In the past few decades, the detection and stimulation of deep brain neurons was based on single-site microwire electrodes using tungsten, gold, platinum, and other metals with high conductivity and chemical stability. Among the earliest researches, Hubel [20] used an electrolytically sharpened tungsten wire insulated to the tip with insulating lacquer to make electrodes for recording action potentials at the level of single cells in 1957. The carbon fiber electrode was first developed in 1970 [21], which consisted of a single carbon fiber insulated by pulled borosilicate glass capillary. These single-site electrodes, or a bundle of several single-site electrodes, have high spatial resolution but limited detection range and efficiency for recording multicellular neural information in primate deep brain. Subsequently, with the progress of MEMS technology, MEAs containing tens to hundreds of recording sites could be fabricated with accurately controlled diameter and spacing to match the size and distribution of neurons in deep brain. They can simultaneously monitor the neural electrophysiological activities of multiple neurons and improve the spatial-temporal resolution of signals. The Michigan probe is one of the most representative devices, which developed a series of commercialized silicon MEA probes for rodents and NHPs [22]. However, due to their high rigidity, brittleness, and more prone to induce immune response, Michigan electrode array for primates is mostly used for intraoperative acute recording and is not suitable for long-term use. To deal with this problem, MEAs fabricated on flexible substrates, such as polyimide (PI) and parylene, were more and more developed, which have lower Young’s modulus compatible with brain tissue, and further reduced the chronic damage of the brain. Although the electrode devices mentioned above have been sporadically tried in human brain, most of them were tentative. The deep brain electrodes for clinical use should pass strict evaluation of safety and efficacy, as well as ethical reviews. At present, only a few types of electrodes have been approved for implanting into deep brain of patients, including stereoelectroencephalography (SEEG) electrodes, DBS electrodes, and some of their variants. Most of them integrated several recording sites with the length of more than 1 mm, which can only record LFPs rather than spikes from single neurons.

**Fig. 2.**
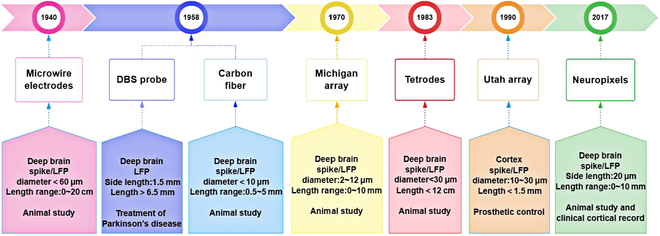
The emergence timeline for different types of primate electrode devices. The upper rectangular box represents the electrode type, while the lower box represents the implanted brain area, detected signal type, electrode size, electrode length range, and application range.

It can be seen that, to achieve high spatial-temporal resolution and long-time detection in deep brain, various electrode array devices have been developed. The electrode forms, the neural recording and stimulating sites, and the implantation methods are quite different depending on the fabrication technics and application scenarios. The common implantation methods include direct implantation, driving propulsion, microneedle guidance, and so on. Optimizing electrodes in material selection, structural design, and electrode size can improve the effectiveness of signal acquisition. Fig. 2 shows the development of different types of electrodes. The electrodes used for the detection of deep brain neural information in primate are displayed below the timeline, followed with their placement position, the detected signal type, the spatial resolution, i.e., the site size, the electrode length range, and the application scenario.

## Types of Primate Neural Information Detection Electrodes

### Metal microelectrodes

#### Single-site metal wire electrode

The single-site metal wire electrode is a metal wire sealed by insulating material except the tip, which is used to record action potential of neurons or LFPs, and also can be used to induce or inhibit action potentials through electrical stimulation. The preparation process of microwire electrodes includes forming electrode tips, connecting electrode wires, insulating, and packaging, which has good material stability and integrity. Generally, tungsten [23], platinum [24], platinum–iridium alloy [25,26], titanium [27], and other materials are used to make metal microwire electrodes, while Teflon, PI, and parylene [28] are used as insulation materials outside the microwire. Inserting microwire electrodes into brain tissue to record the electrical activity of a single neuron is widely used in research on NHPs.

Single-site tungsten wire electrode is often used for functional localization during surgery before implanting DBS electrodes in patents with Parkinson’s disease, which can accurately find the target nucleus. Jamali et al. [29] implanted tungsten electrodes into the dorsomedial prefrontal cortex of the human body to predict their reasoning ability to others. The tungsten wire typically has a diameter of about 200 μm and a length of more than 20 cm, which can be fixed on the human brain stereotaxic framework for precise implantation into the target brain region. The preparation process of tungsten wire electrode includes cutting, tip corrosion, cleaning of tungsten wire, coating of parylene insulating layer, and assembling PI or stainless steel cannulas on tungsten wire. The modification of highly conductive materials on the electrode tip can enhance the detection and stimulation effect of the electrode. Therefore, Keefer et al. [30] used electrochemical technology to coat carbon nanotubes (CNTs) on the tungsten wire electrode, which decreased the impedance, increased charge transfer, and thus enhanced the effect of neural recording and electrical stimulation in primates. PI tubes and stainless steel tubes coated outside tungsten wire can effectively shield noise and assist electrode implantation. The single-site tungsten wire electrode can record action potentials from single neurons, but cannot achieve multi-channel neural information detection, so it is very important to innovate and fabricate multi-sites on the microwire.

#### Tetrode

**Fig. 3.**
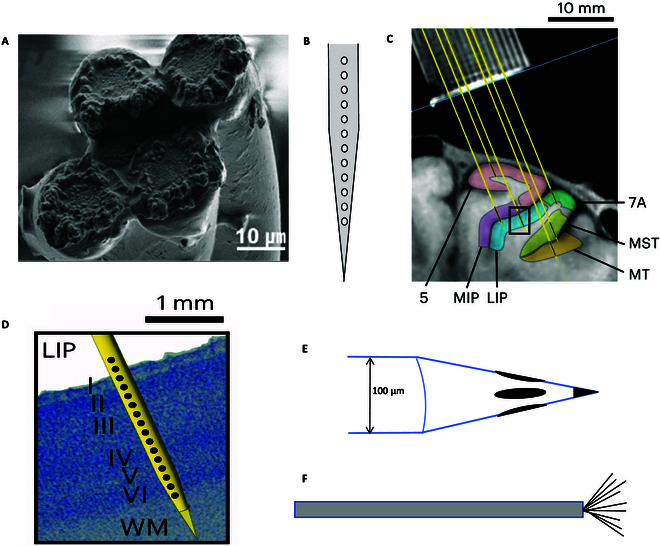
Metal microelectrode. (A) Improved twisted wire tetrodes. Reproduced with permission from [32,157]. Copyright 2018, Experimental Neurobiology. (B) FHC laminar electrode array (schematic diagram). (C) V-probe implantation sites in the monkey’s cortical areas: MT, MST, 7A, 5, MIP, and LIP and (D) the location of V-probe recording channels relative to the cortex. Reproduced with permission from [43]. Copyright 2024, Nature Neuroscience. (E) Thomas recording tetrodes electrode (schematic diagram). (F) Behnke–Fried electrode tip (schematic diagram).

Stereotrode [31], which was initially composed of 2 twisted wires, can separate a single neuron from the recorded data, and soon the tetrode composed of 4 metal wires was invented. Then, the improved twisted wire tetrodes (TWTs) appeared, which can record the signals of neuronal groups and facilitate spike sorting to distinguish the signals of multiple nearby neurons (Fig. 3A). TWTs are helpful for its application in the macaque brain. Kapoor et al. [32] developed a new type of tube tetrodes. They are wound by 4 ultrathin NiCr wires (diameter, 12 to 25 μm), which are sleeved into PI tubes, and finally sleeved into stainless steel tubes. Compared with ordinary tetrode, their tensile strength is higher. It was implanted into the temporal lobe of the macaque brain with a microdriver. The signal quality obtained by it was very good, and it could record multiple single-unit signals. The ultrathin diameter of the tetrode can cause little damage to the brain.

#### Multi-site metal wire electrode

Multi-site metal wire electrodes have been widely used in primate neural information detection for a long time. Thomas recording microelectrode [33] appeared in 1983. It is made of platinum tungsten material and insulated by quartz glass. It has high impedance stability, but there are few electrode sites. The electrode length is customizable, with available diameters of 40, 60, or 80 μm. Therefore, the improved Thomas recording tetrodes electrode appeared in 1987 [34] (Fig. 3E), which arranges 4 metal sites in different directions of the electrode and insulates each other through quartz glass, so it has the advantage of low impedance and high signal-to-noise ratio, and can adapt to brain regions with different cell densities. Subsequently, a further improved Thomas recording heptodes electrode [35] appeared, increasing the number of metal sites to 7, further enhancing the electrode function and enabling spike sorting. The newly developed Thomas recording 3D-heptodes electrode [36,37] has 7 sites arranged at 3 different heights, so it can record 3 different layers of neural structure records. The diameters of microelectrodes on Thomas recording tetrodes, Thomas recording heptodes, and Thomas recording 3D-heptodes are all 95 μm. Thomas recording linear stainless steel multitrodes [38,39] are increased to 8 channels for the acute recording of large- and medium-sized animals, which can be made into different lengths. The 5 types of electrode devices mentioned above are capable of multi-neuron recording and can be customized in length to accommodate varying implantation depths. The electrodes have the characteristics of low impedance, low noise, high signal-to-noise ratio, and little damage to brain tissue. Tamura et al. [35] used Thomas recording heptodes electrode to stimulate and record the TE region of the primate inferior temporal cortex, and found that neurons near the TE area showed a weak positive correlation in terms of stimulation preference.

Many different kinds of commercialized multi-site metal electrodes can record neuronal signals in deep brain of NHPs, providing a wealth of options to meet various research needs. For example, multi-site metal wire electrodes can be applied in the study of spatial cognition. These electrodes can record neural signals from multiple deep brain locations such as hippocampus and entorhinal cortex, which helps in understanding the neural mechanism in processing spatial information. AXIAL array is a kind of multi-site laminar electrode array provided by FHC Inc. (Fig. 3B). Killian et al. [40] used aforementioned laminar electrode array mounted on tungsten microelectrode (12-site, 30 μm diameter, 150 μm spacing) in 2012 to record spike and LFP signals in the entorhinal cortex of primates, proving that there are grid cells in the entorhinal cortex of primates. Meister and Buffalo [41] used the laminar electrode array of 13 channels in 2018 and found that primate entorhinal cortex neurons can complete memory and motor planning functions by coding location. Chiang et al. [42] used tungsten microelectrodes (FHC Inc.) to explore the cognitive strategies of monkeys. V-probe is a multi-site linear electrode based on stainless steel needle provided by Plexon Inc. It can integrate the fluid channel of drug delivery and the light path of optogenetic stimulation. It is mainly used in the acute research of large animals such as primates. Mendoza-Halliday et al. [43] use V-probe (Fig. 3C and D) to record electrical activity and optogenetic response in NHP. The Deep Array is a high-throughput high-density electrode based on stainless steel or tungsten provided by Diagnostic Biochips Inc., which is targeted at chronic and acute recordings in deep brain regions of large animals such as NHPs. Abbaspoor and Hoffman [44] used the 128-site Deep Array with a single column linear span for recordings in the macaque hippocampus to study circuit dynamics of superficial and deep CA1 pyramidal cells. Because stainless steel or tungsten substrates are durable and can be reused, they are expected to be widely used in the field of acute recordings in primate deep brain.

However, multi-site metal wire electrodes have some limitations, as long-term implantation remains a challenge due to electrode degradation, brain tissue encapsulation, and neural signal recording instability, which can affect signal quality over time [45]. Additionally, inflammatory responses and glial scarring around the implantation site can further degrade recording performance [46].

#### Microwire array electrodes

One limitation of abovementioned devices is that all the electrode sites are linearly distributed longitudinally along the single microwire needle, while in many applications, it is necessary to integrate multiple wires in parallel to cover larger area laterally. Tungsten, platinum iridium alloy, and stainless steel microwires could be assembled manually or semiautomatically to form such array, which integrated tens to hundreds of microwires. Each microwire is insulate except the tip, with the diameter of 20 to 30 μm and the length of less than 10 mm. Since the microwires are thin and soft, biodegradable materials such as polyethylene glycol (PEG) are coated on the wires to avoid deformation, protect the tips, and assist implantation. Once the metal wires were inserted into the brain tissue, the material of PEG would dissolve to expose the wire tip for electrophysiological recording on single-neuron level. Nicolelis et al. [47] implanted microwire array composed of hundreds of stainless steel microwires into the monkey cerebral cortex for long-term multi-site recording. High-quality neural action potentials were recorded in awake, behaving monkeys, and the electrodes were still effective 18 months after implantation. The microwire arrays provided low-cost and high-efficient tools for investigation of motor, perception, and learning in primates and promoted the development of neuroprosthetic devices. However, due to the short length of the microwire array electrode, when used on primates, its application scope is limited to cortex. The situation is improved by the appearance of the metal microwire bundle electrodes, which have a longer length and can detect in deeper brain depths.

#### Metal microwire bundle electrode

Integrating many metal wires into a microwire bundle can detect more neuronal signals in deeper brain. To track the behavior of awake monkeys for a long time, McMahon et al. [48] made a kind of driven and long-term implanted microwire bundle electrode. The microwire bundle electrode entered the brain through a PI catheter passing through the dura mater and was guided to the monkey’s infratemporal cortex through an adjustable microdriver. Subsequently, the activities of single neurons were recorded and separated to study the visual response of monkeys for a long time. McMahon et al. also used the microwire brush array (Microprobes Inc.), which can also perform long-term recording. The array integrates multiple microwire into a microfibril tube and can be implanted into the brain to record and stimulate small neurons below the cortical surface. Courellis et al. [49] used 64-channel microwire brush array to record the neural activity generated by the hippocampus of freely moving marmosets when exploring the environment. Worrell et al. [50] used a depth electrode composed of a microwire bundle and clinical macroelectrode (Adtech Inc.) to record high-frequency oscillations in the human temporal lobe, and then determined the epileptic brain region. Metal microwire bundle electrode not only is widely used in monkey brain research but also has important applications in human. Behnke–Fried electrode (Fig. 3F) realized the recording of human brain extracellular neurons, but its platinum wire electrode tip is fragile, and excessive stretching during surgery will cause electrode breakage leading to the loss of single-neuron signal [51]. Misra et al. [51] implanted Behnke–Fried depth electrode and microwire bundle electrode into the temporal lobe of epilepsy patients to perform single/multi-unit recordings, and found that noise, motion artifacts, and other problems would be generated during the recording process. At present, the acute recording method of rigid electrodes has been widely used, but its advantages and limitations coexist. It is vulnerable to the relative movement between respiration and brain tissue, which leads to the growth of glial cells and wraps the device, so its service life is short [52]. Compared with rigid electrodes, the abovementioned metal microwire bundle electrode can provide relatively stable detection. Although the metal microwire bundle electrode can be placed at different depths, it requires a rigid catheter to introduce it into the deep brain region due to its low flexural rigidity, which may produce greater damage.

### Silicon-based microelectrodes

Different from the metal wire electrodes and their traditional processing and assembly methods, silicon-based microelectrodes are fabricated by micro-nano machining technology on silicon wafers. Through surface and bulk micromachining, such as thin-film deposition, photolithography, and silicon etching, the shape and size of silicon probes and microelectrode sites could be accurately defined with the machining precision of less than 1 μm. More importantly, they can integrate tens to hundreds of detection channels in a tiny device. Utah array and Michigan probe are 2 typical silicon-based microelectrodes. Although most of them can only be applied to cortical detections for primates, their importance cannot be ignored.

#### Utah electrode array

**Fig. 4.**
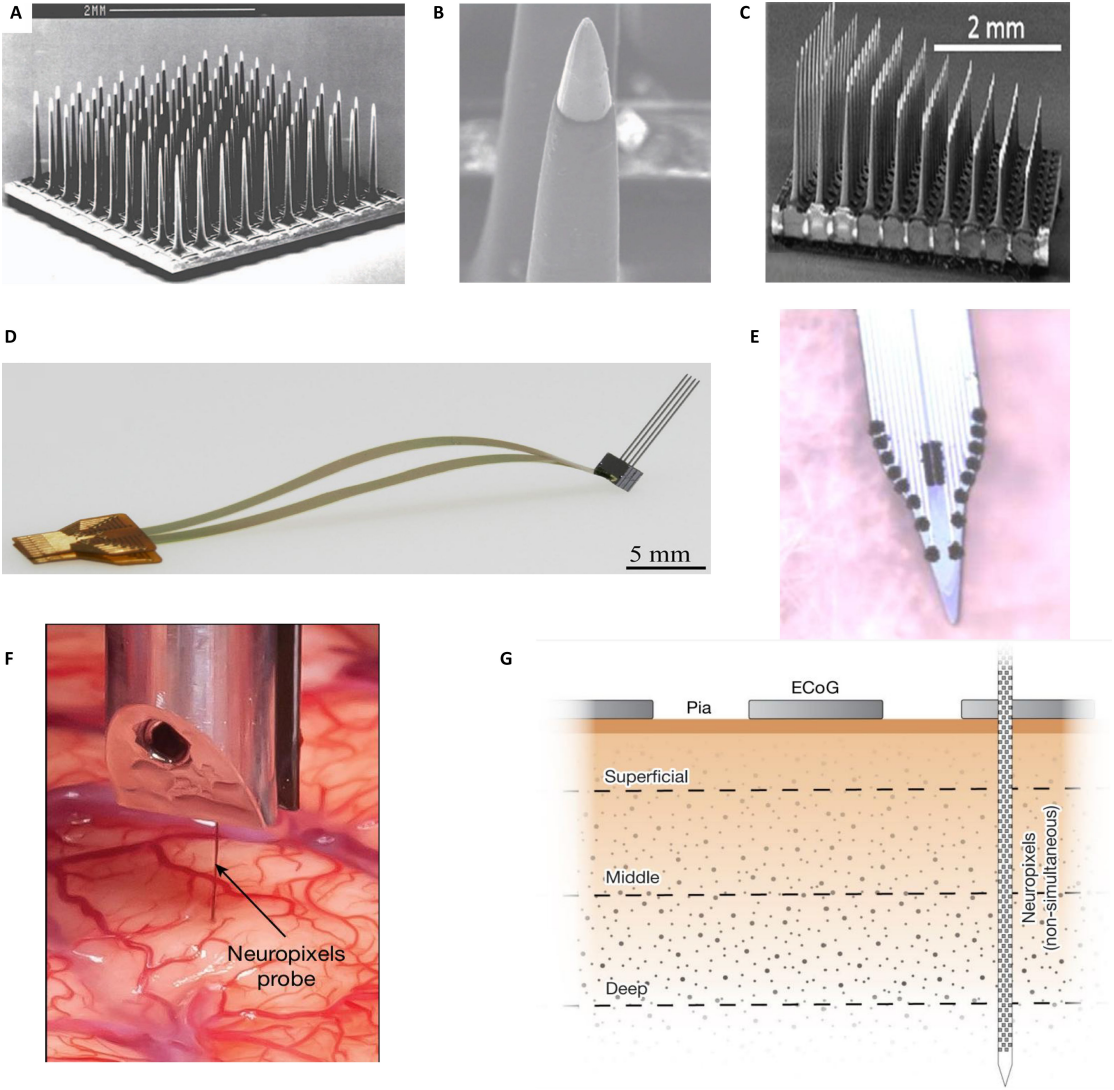
Silicon-based microelectrode arrays. (A) Utah electrode array is fabricated on silicon substrate and (B) magnified view of the Utah electrode tip. Reproduced with permission from [53,56]. Copyright 2021, Journal of Neural Engineering. (C) The electrode length of the Utah slant array has a linear distribution. Reproduced with permission from [69]. Copyright 2017, Journal of Neuroengineering and Rehabilitation. (D) 3D electrode array assembled from Michigan electrodes. © IOP Publishing. Reproduced with permission from [71], 2017, Journal of Neural Engineering. (E) Silicon-based dual-mode implantable microelectrode array. Reproduced with permission from [75]. Copyright 2016, Biosensors and Bioelectronics. (F) Implanting Neuropixels into the cortex of human brain and (G) Neuropixels and macroelectrode position relative to the human brain. Reproduced with permission from [82]. Copyright 2024, Nature.

Norman and colleagues [53–56] from the University of Utah created a silicon-based Utah array in 1990s (Fig. 4A and B). It will etch the cut silicon column to form a silicon-based array [57], which is very important in the study of NHPs and human cortex [58]. The silicon-based Utah electrode array has an electrically active site at the tip of each probe. In addition to these sites, the entire array is insulated with biocompatible materials, and the probe size can be configured to be constant [54,59] or different lengths [60,61] according to different uses. The Utah array is widely used in clinical BCI research and other fields. It can also record the activity of individual neurons of patients, decode handwriting, control artificial limbs, and provide sensory feedback [62–67]. Fernández et al. [68] implanted 96-channel Utah electrodes in the visual cortex of blind people, which can make patients’ vision produce weak perception through electrical stimulation. Based on traditional Utah electrodes, researchers have developed many new Utah array variants. Shandhi et al. [58] fabricated 9 active sites along each probe and around the silicon-based Utah electrode, making the channel density 9 times that of the Utah array. The electrode length of the Utah slant array (Fig. 4C) has a linear distribution [69]. It can cover the cross-section of the neural bundle and can be used for human and animal research.

The limitation of Utah electrode array is that the length of each electrode is about 1.5 mm, which is confined by the thickness of silicon wafer. It could only be implanted in the cerebral cortex and capture action potential signals from neurons in cortical surface area. The detection depth is still not enough to obtain the signals from neurons in the folded cortex and deep brain. Compared with the Michigan electrode described below, the Utah array consists of 100 rigid conductive silicon needles to form 3D electrodes, but only receives signals from the tip of each electrode, limiting the information obtained at one time.

#### Michigan electrode array

Wise et al. [70] from the University of Michigan first developed the silicon-based Michigan electrode array by micromachining technology. The device consisted of multichannel thin-film microelectrodes insulated by deposited dielectrics and supported by one or several planar microprobes. Compared with the silicon-based Utah array, the silicon probes of the Michigan electrode array are thinner, and there are multiple electrode sites on each probe, which can realize stereo recording. The length of the probes can be fabricated into a few millimeters to centimeters, which is longer than that of the Utah array, and is more suitable for recordings in deeper areas of the brain. Michigan electrode array has been widely used in rodents and cats. However, because the Michigan electrode is fragile and easy to break, it is necessary to remove the dura mater during the implantation process, and it is less applied in the deep brain of NHPs. Barz et al. [71] proposed a 3D electrode array assembled from Michigan electrodes and successfully carried out NHP experiments (Fig. 4D). They implanted the electrode array into the pre-supplementary motor area F6 of rhesus macaques for hand grasping tasks.

The Michigan electrode has also been expanded to many variants. Vector Array, which consists of a 32-site Michigan silicon probe and a stainless steel protective sleeve, was designed for acute deep brain detection in NHPs. It can be implanted to the maximum depth of 80 mm for deep brain nucleus to record extracellular neuronal activities and perform electrical stimulation. When implanted, the silicon-based electrode is embedded in the sleeve, which can reduce the damage to the electrode array and extend the service life of the probe. This electrode design has further promoted the detection capability of Michigan electrodes in the deep brain region of NHPs. The Matrix Array 3D recording electrode with iridium site is a 3D device designed based on the Michigan electrode array, which can be used for acute and chronic experiments in NHPs. The electrode can record and stimulate a large number of neurons in the brain and spinal cord in 3D space, and the depth can reach 10 mm. The electrode array covers a large tissue area, has high channel density, and is robust.

Silicon-based Michigan electrodes have multiple sites on each electrode, which can increase the throughput of signal acquisition from the cortex to the deep brain region. The silicon probe is thin and fragile, as mechanical stress or long-term implantation can lead to fractures [72], which can affect their functionality and longevity and limit the depth of its implantation into the brain. Therefore, assisted implantation with the outer sleeve can reduce the damage to the electrode array, but it will increase the injury to the brain tissue. Furthermore, silicon-based electrodes can induce immune response, potentially reducing their stability over time [73]. Michigan array has a high density of implanted sensors and a higher spatial resolution than microwires, and it can obtain signals at different depths. With the advent of the Vector Array and 3D Matrix Array, the recording depth and efficiency have been further improved.

#### Other silicon-based microelectrodes

In addition to the Utah electrode array and Michigan electrode array, researchers have also developed many other new silicon-based microelectrodes through MEMS, complementary metal oxide semiconductor (CMOS), and other fabrication processes. Sauter-Starace et al. [74] sputtered Ti and TiN on silicon-on-insulator (SOI) wafer to form the conductive layer and then released the silicon probe. The probe adopts the MEMS wafer processing technology and then modifies the recording site with CNT to record primate seizures. The implantation depth can reach 3 mm at most. In the process of microelectrode fabrication, the site surface modification materials can increase the specific surface area of the electrode and reduce the electrode impedance. The above methods of modifying CNTs provide a new perspective for modifying the electrode materials.

Zhang et al. [75] designed a dual-mode silicon-based implantable MEA, which is a linear electrode similar to the Michigan structure. As shown in Fig. 4E, the single probe of the device has 16 recording sites, with the probe length of 25 mm and a cross-section of 30 μm × 300 μm. The microelectrode surface is modified with platinum black nanoparticles and Nafion, which can be used for electrophysiological recording of spikes and LFPs, and also for electrochemical detection of neurotransmitter dopamine. The probe was implanted into the brain of cynomolgus monkey from cortex to striatum, and the dual-mode signals were concurrently recorded in real time. The MEA has high spatial resolution, high detection sensitivity, and dopamine selectivity [76,77].

In order to dramatically increase the microelectrode density on one probe, CMOS technology was more and more used in fabrication of neural electrode arrays. Fabrication process of CMOS technology is compatible with MEMS technology, and the feature line width can be reduced from a few micrometers to 100 nm, which effectively increased the number of electrodes per unit area. At the same time, the neural signal processing circuit can be integrated on the electrode device. Jun et al. [78] have made a Neuropixels electrode integrating 960 recording sites on a single, 10-mm-long, nontapered silicon shank with 70 × 20 μm cross-section based on CMOS process. The device has been used in the recording of deep brain neural information in rodents [79] and NHPs [80]. For human applications, the probe thickness was increased to 97 to 100 μm, which allowed for tolerance of greater mechanical forces for larger brain recordings, and less easy to break during brain surface fluctuations induced by respiration and blood pressure. Paulk et al. [81] used Neuropixels electrode for cortical neural recording in human participants during intraoperative neurosurgical procedures. Over 200 well-isolated cortical single units were simultaneously recorded at unprecedented spatiotemporal resolution. Leonard et al. [82] used Neuropixels electrode to detect the auditory region of the superior temporal gyrus of the human cerebral cortex (Fig. 4F and G), and thus found the coding of speech by single neurons. The appearance of this high-density probe provides a tool for further understanding of human-specific cognitive processes and their dysfunctions. Although the linear silicon array has high spatial-temporal resolution and has made important progress in the recording of human single neurons, there are still limitations. For example, it cannot reach nucleus deeper than 10 mm under cortex, and only one shank probe is used for neural signal recording, which leads to the inability to identify potential neural circuits. In addition, significant changes in spike waveforms and brain tissue damage would be caused by brain movement relative to the probe due to breathing and other factors. The development of this probe toward deep brain recording also needs improvement on fabrication process and probe material.

Although silicon-based electrodes can obtain high-quality neural signals, due to their fragility and ease of breaking, some electrodes are limited in implantation depth and are difficult to reach deep brain areas of primates, such as basal ganglia and hippocampus. These areas are crucial for motor control, cognition, learning, and memory. So, it is necessary to design, fabricate, and apply other protypes of new electrodes.

### Flexible multichannel electrodes

To reduce the immune reaction caused by the relative movement between the rigid electrodes and the brain, it is very important to design flexible electrodes for long-term recording. Flexible materials such as parylene-C and PI are commonly used as flexible substrates, which incorporated multichannel thin-film MEAs fabricated by MEMS technology. They usually have good biocompatibility, softness, and mechanical properties very similar to neural tissue [83]. A flexible multi-channel MEA can produce less immune response, but because of its soft characteristics, it is difficult to insert into brain tissue. A variety of insertion methods need to be developed to ensure that the inserted electrode causes less damage to brain tissue and is accurately placed in the correct position.

**Fig. 5.**
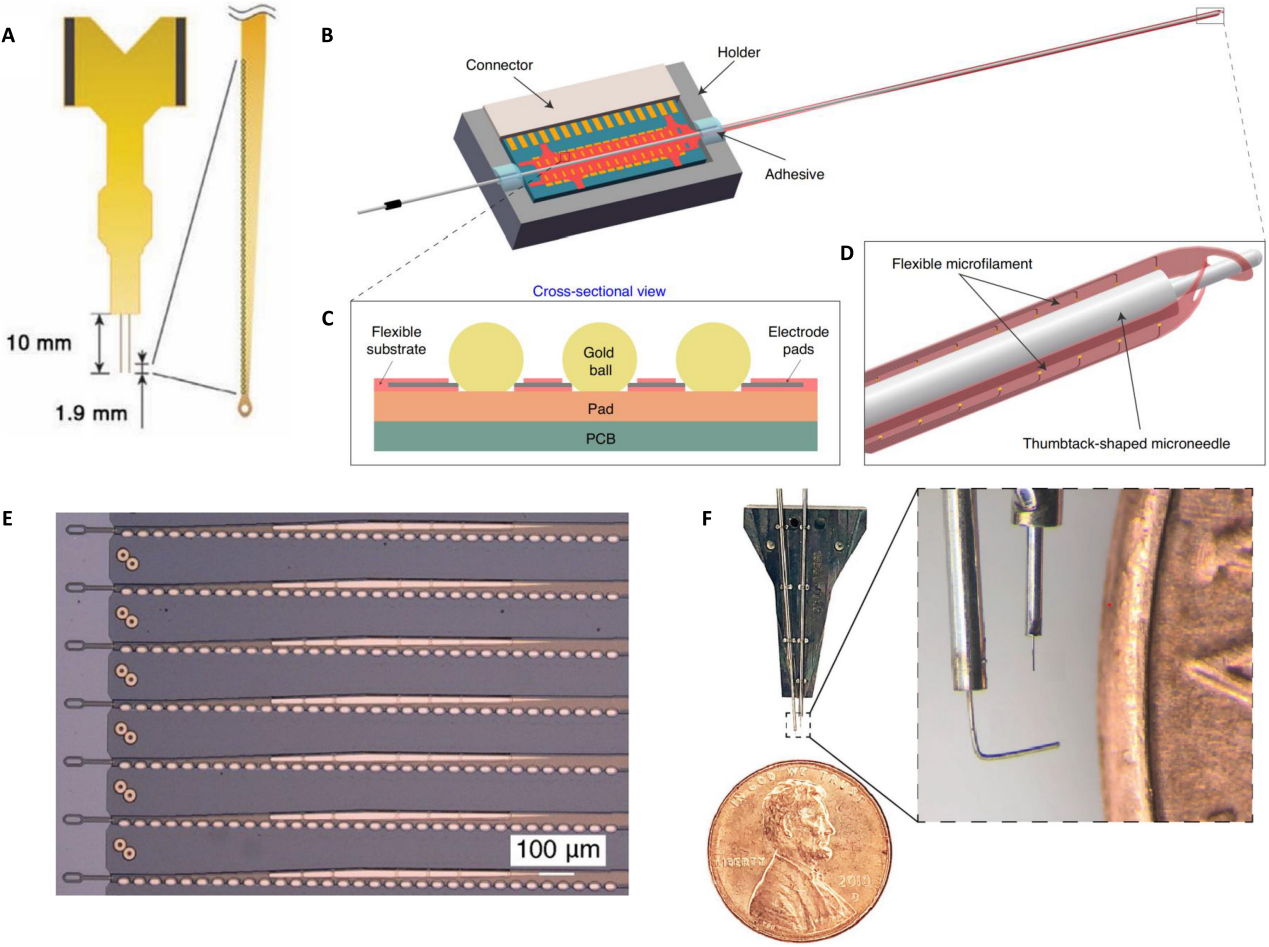
Flexible multi-channel electrode arrays. (A) Mechanically robust ultraflexible electrode array and its tip enlarged image. Reproduced with permission from [84]. Copyright 2023, Advanced Science. (B) Schematic of an electrode assembled with flexible microfilaments and rigid microneedles. Reproduced with permission from [85]. Copyright 2023, Microsystems and Nanoengineering. (C) The electrode and PCB are connected using gold balls. Reproduced with permission from [85]. Copyright 2023, Microsystems and Nanoengineering. (D) Schematic diagram of the tip of “needle filament tube”. Reproduced with permission from [85]. Copyright 2023, Microsystems and Nanoengineering. (E) Neuralink flexible implantable electrode array with 3,072 electrode sites and (F) size comparison of Neuralink guiding needle and a coin. Reproduced with permission from [89]. Copyright 2019, Journal of Medical Internet Research.

Tian et al. [84] have made a new 128-channel mechanically robust ultraflexible electrode array (MERF), as shown in Fig. 5A. It uses flexible PI as the substrate to create 2 implantable shanks, each with 64 microelectrode sites. Electrode sites are arranged at the edge of each shank to allow closer proximity to neurons. Its maximum implantation depth is 1.9 mm, which allows for long-term recording of the cerebral cortex in NHPs. However, this implantation depth is still insufficient for deep brain nucleus. Wang et al. [85] made a flexible 16-channel electrode array based on PI microfilament with the length of 60 mm and designed a “needle-filament-tube” implantation method (Fig. 5B). The electrode is connected to the PCB using gold ball bonding, ensuring a stable and reliable electrical connection. This method enhances durability and performance for neural signal recording (Fig. 5C). Two parallel flexible microfilaments with better biocompatibility and lower flexural rigidity are wrapped on both sides of a rigid tungsten microneedle, and then they were together inserted into a stainless steel guide tube (Fig. 5D), which helps puncture the dura, protect the microelectrodes, and shield noise. Driven by the rigid microneedle, the totally 32-channel microelectrodes were implanted into the dorsal premotor cortex in the macaque brain to record task-related neuronal activities. However, the guide tube and microneedle have to be retained during the recording process, which only applies to head-fixed animals, and it is also difficult to give full play to the advantages of the flexible microfilaments, limiting its application in chronic recording.

Therefore, it is very important to design an implantation method that can help insert the flexible electrode into the deep brain and then the auxiliary rigid object can be remove. Liu et al. [86] developed a 1,024-channel Neuroscroll probe for rodents and NHPs. It is fabricated by photolithography, metal deposition, and dry etching on PI thin films, and then rolled onto tungsten wire to produce MEA. The length of the Neuroscroll probe is flexible and adjustable, and it remains functional after repeated bending, showing high mechanical stability. The experimental results showed that the probe provided high chronic stability and reliable recording for up to 105 weeks. Its modular design and high-density flexible cold bonding (FCB) connection method make it easy to be compatible with miniaturized electronic equipment. However, defects such as microparticles and scratches can lead to yield loss, and using an industrial-level clean environment can reduce these defects and improve yield.

Researchers have also developed many commercially available flexible base electrodes, such as the 24-channel MicroFlex array, which can be used to record and stimulate neural signals. It uses PI as the substrate, and the multi-site electrode array with an electrode diameter of 15 to 100 μm can be placed in deep brain regions such as the hippocampus [87]. It can record single-unit, multi-unit, and LFP signals. Because of its flexibility, it can minimize tissue damage. However, brain implantation requires an insertion needle to help, so the issue of insertion trauma needs to be addressed [87,88]. While the recent Neuralink flexible implanted electrode array takes PI as the substrate and contains 3,072 gold electrode sites [89] (Fig. 5E and F), the electrode is guided by tungsten needle, and a light module at the tip of the auxiliary inserter guides the electrode to the correct position. So far, the electrode has been applied in NHPs and clinical research [90].

### Deep brain electrodes related to clinical major brain diseases

In the past decades, advances in material processing have improved the resolution of neural recording devices. At the same time, the development of microsystem fabrication, including MEMS, CMOS, laser cutting, and other technologies, has innovated the electrode form and structure. It has become possible to detect the electrophysiological activity of neurons in the deep brain of NHPs. In animal models, through the analysis of the action potentials of thousands of neurons, we can obtain a rich understanding of neural activities based on the collection of neurons such as sensory, motor, and cognitive [80]. However, further development is needed in human application. In clinics, recording the activities of single neurons in the human brain has become more and more frequent [91–94], which were conducted during surgery for Parkinson’s disease, epilepsy, and other neurological diseases [95,96]. In addition to the Utah electrode, the DBS electrode and SEEG electrode are also used in the clinic.

**Fig. 6.**
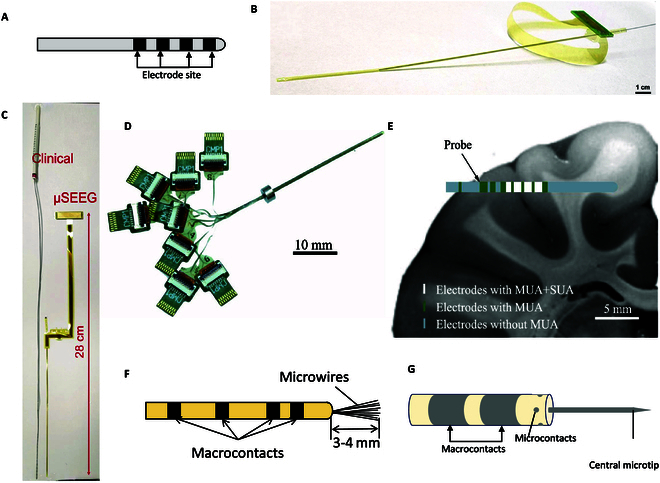
Deep brain electrodes related to major clinical brain diseases. (A) DBS electrode applied in clinical study (schematic diagram). (B) A 128-channel thin-film electrode and (C) comparison with clinical electrodes. Reproduced with permission from [104]. Copyright 2024, Nature Communications. (D) SEEG electrode and (E) implantation at 2 cm of the monkey’s brain. Reproduced with permission from [105]. Copyright 2016, Journal of Neural Engineering. (F) iEEG electrode has large contacts and microwires protruding from the tip (schematic diagram). (G) AlphaProbe schematic. The electrode consists of 2 macrocontacts, 4 microcontacts, and 1 central microtip (Alpha-Omega Inc.).

DBS electrodes are clinically used to treat Parkinson’s disease. They are usually implanted into the deep brain nucleus such as STN, the pallidus nucleus, or the ventral intermediate nucleus of the thalamus (Vim). In Parkinson’s patients, bilateral DBS has been associated with significant improvements in motor complications after 6 to 12 months of therapy [97]. Through follow-up evaluation of patients 3 to 4 years later, some issues were identified, such as surgical complications, wire breakage, skin corrosion or infection at the battery site, and cognitive impairment. Clinical data show that DBS can significantly improve quality of life and reduce drug dependence, but its long-term effects require further research. DBS electrodes can be used in the treatment of not only Parkinson’s disease but also other neurological diseases, such as epilepsy, obsessive–compulsive disorder, and Alzheimer’s disease [98]. For individuals with drug-resistant epilepsy, DBS targeting mesial temporal lobe epilepsy in 4 patients has demonstrated a mean seizure frequency reduction of 75% in some studies [99]. DBS electrodes have potential risks including the occurrence of seizures after placement, which, although uncommon, typically manifest early in the postoperative period [100]. The existing DBS electrode [101] (Fig. 6A) has made important progress in simultaneous electrophysiological recording and stimulation, but it is unable to detect single-cell level neural information.

Stereotactic implantation of depth electrodes is the preferred method for detecting electrophysiology activity and localizing conditions such as epilepsy [102] in many surgeries. Compared with EEG, the depth electrode has a relatively higher spatial resolution, which can be used for the SEEG study of epilepsy and localization of epileptic focus. Suthana et al. [103] implanted deep electrode (Adtech Inc.) with platinum contacts in the subject’s deep brain hippocampus and entorhinal cortex for recording and stimulation to verify their ability to convert long-term memory when learning spatial location. Lee [104] developed a 128-channel thin-film electrode (Fig. 6B and C) for patients with epilepsy and other neurological disorders, capable of precisely recording and stimulating lesion sites at depths of up to 10 cm in the brain, capturing the action potentials of individual neurons, and wirelessly detecting brain activity. This innovation overcomes the limitations of SEEG electrodes, such as their relatively large size and imprecise localization. Pothof et al. [105] proposed a new SEEG electrode (Fig. 6D) fabricating process, using PI as the substrate of the electrode, rolled it into a cylindrical shape as the electrode probe, and implanted it at 2 cm of the monkey’s brain (Fig. 6E). This electrode has potential value in clinical application. Researchers have made numerous improvements and developed various other types of electrodes based on the form of SEEG electrode for reference. Quiroga [106] used an intracranial electroencephalographic (iEEG) electrode (Fig. 6F) that protrudes microwires from the tip of the conventional SEEG electrode to record LFP and spike activity. Ekstrom [107] implanted iEEG electrode devices into hippocampus of epileptic patients and found that neurons respond to specific spatial locations that resemble place cells in rodents. Jacobs [108] used similar technics and identified neurons exhibiting grid-like spiking patterns in entorhinal cortex of the human brain.

At present, some clinical treatment-related electrodes for major brain diseases have been developed, such as AlphaProbe (Fig. 6G), which has 7 sites on the electrode and can record and stimulate. There is one central microelectrode, 2 macroscopic electrodes, and 4 lateral microelectrodes on both sides of the electrode axis, which are stereotactic electrodes with high spatial resolution. There have been many new concept electrodes potentially applied to human beings. Pothof et al. [109] designed an SEEG neural probe based on a PI layer sandwich. This electrode is a new concept electrode, which can be used to diagnose and treat neurological dysfunction and achieve precise brain positioning in the future.

## Neurotransmitter chemical detection electrodes

The traditional microdialysis method using microdialysis probes in the detection of neurotransmitters in humans and animals has been widely recognized. It can sample and analyze the concentration changes of chemical substances in specific areas at a certain time interval in the body to provide valuable information about the dynamic changes of neurotransmitters. However, due to the large size of the probes, which is usually several milimeters in diameter and length, the spatial-temporal resolution of microdialysis is low, and the sampling area is large. It cannot accurately analyze the rapid and local neurotransmitter changes. To solve this problem, a neurotransmitter electrochemical detection electrode came into being. Compared with microdialysis, electrochemical microelectrode has higher spatial-temporal resolution and can detect the rapid changes of neurotransmitters on the millisecond time scale and the micrometer spatial scale. This advantage makes electrochemical microelectrodes an increasingly important tool in neuroscience research, especially in applications that require high-resolution detection of neurotransmitter dynamics. Neurotransmitter chemical detection electrode is used to monitor the concentration changes of neurotransmitters (such as dopamine, glutamate, and so on) in the organism. These electrodes are usually composed of CNTs, carbon nanofibers, or other conductive materials and are designed into small and highly sensitive structures. The working principle of these electrodes is based on electrochemical methods. By introducing specific biomolecules (such as enzymes or receptors) on the electrode surface, they can selectively react with target neurotransmitters, and will produce current or voltage signals, the amplitude of which is proportional to the concentration of the target transmitter. Neurotransmitter chemical detection electrodes are usually used in neuroscience research and the diagnosis and treatment of major brain diseases. Their high sensitivity and selectivity make them an important tool to study the dynamic changes of neurotransmitters.

### Carbon fiber electrodes

In recent years, flexible carbon-based microfiber electrodes have developed rapidly. Its small size and flexible characteristics not only reduce the foreign body reaction when implanted into the brain but also improve the signal-to-noise ratio, resolution, and flexibility of the electrode [110], which can be used for neural recording and stimulation. At present, there are 3 main forms of carbon-based microfibers for neural recording and stimulation: carbon fibers, CNT-based fibers, and graphene-based fibers, which can detect rapid changes of dopamine levels in the brain.

The carbon fiber electrodes are small in size, resulting in a negligible immune response at the implantation site. Their porous structure provides a large surface area [111] and allows for chronic detection and stimulation. Sands et al. [112] used carbon fiber electrodes to detect dopamine release in the striatum of the human brain, and then found the relationship between dopamine and reward and punishment prediction. Carbon fiber electrodes coated with materials such as conductive polymer or iridium oxide on the surface can improve the charge injection capacity for neural stimulation [113–117], but the degradation and delamination of the coating material will limit the life and long-term use of the electrode [118]. The second kind of CNT-based fibers has a high-quality specific surface area, high conductivity, and good electrochemical characteristics, so CNTs are used to improve the electrode performance of neural stimulation and recording [30,119,120], which can be used for chronic recording. The graphene-based fibers have a large effective surface area, good biocompatibility, and sufficient mechanical strength. They can be inserted into the brain without additional help [121,122]. Cass et al. [123] used Nafion-coated carbon fiber electrodes to detect the changes in dopamine levels in the brain of monkeys with striatal injury (that is, monkeys with Parkinson’s disease). The diameter of these flexible carbon-based microfiber electrodes is only tens of micrometers, causing minimal damage to biological tissues. However, their mechanical strength is small, making them susceptible to breakage during implantation and chronic recording. Therefore, the mechanical strength of the electrode can be improved by coating it with water-soluble sucrose and implanted into neural tissues [124].

The technology of assembling a single carbon-based microfiber electrode into a high-density MEA is under development and has been used in rodents or birds. Patel et al. [125] welded carbon fibers to polyimide printed circuit board (PCB) and coated the outer layer with epoxy resin, but the electrode was made by hand, which would cause fiber fracture, insufficient exposure, and other problems. Gillis et al. [114] designed 2 kinds of carbon fiber array manufacturing processes, 3D printing fixed fiber and depositing indium on carbon fibers and welding it to PI at low temperatures. Massey et al. [126] fixed carbon fibers onto silicon-based substrates fabricated by the micromachining process, but the fibers are very easy to gather together. Guitchounts and Cox [127] fabricated a plastic block containing 64-channel carbon fiber electrodes through 3D printing and led it out with a 70-pin Hirose connector. These electrode preparation methods provide useful inspiration for the innovative fabrication of deep brain electrodes in NHPs. In the future, carbon-based microfibers can be guided by metal wires or catheters, coated with biocompatible soluble substances, or assembled into high-density microarrays and implanted into the deep brain to improve electrode performance.

### Ceramic-based electrodes

The substrate material of ceramic-based electrodes is usually made of alumina (Al_2_O_3_) and other ceramic materials. The electrode sites for chemical detection are made of metals such as platinum and chromium. Modification of enzymes on ceramic-based electrodes can detect the changes in glutamate and some electroactive monoamines in the brain [128]. Compared with traditional metal electrodes, ceramic-based electrodes have some advantages, such as better corrosion resistance and chemical stability, as well as good biocompatibility and long-term stability. Stephens et al. [129] made ceramic MEA (the site dimensions are 15 μm × 333 μm) modified with enzymes to detect neurotransmitters in the brains of NHPs. Hampson et al. [130–132] used the ceramic MEA of the University of Kentucky (the site dimensions are 22 μm × 80 μm [133]) to explore the firing patterns of the prefrontal cortex in NHPs. Ceramic-based electrodes may be applied to the detection of deep neural information in the human brain in the future [134].

### Other electrochemical electrodes

Other electrodes with different kinds of substrate, such as silicon-based electrodes, can selectively react with target neurotransmitters by introducing specific enzymes or receptors on the electrode surface. The silicon-based electrode sites are composed of metals such as platinum and gold. The dual-mode electrode described in other silicon-based microelectrodes can also be used for neurotransmitter detection.

Because the electrode types discussed in this paper are applied to the deep brain neural information detection of primates, the scalp electrode EEG and the electrode attached to the surface of the cerebral cortex such as ECoG are not within the scope of the above examples. The parameters of the primate deep brain neural information detection electrode described in this chapter are shown in Table 1.

**Table 1.** Comparison of electrode parameters for neural information detection in some primates

Electrode type	Number of sites	Implantation depth	Size	Record spike/LFP	References
Thomas recording microelectrode	1	0–20 cm	Diameter: 40, 60, or 80 μm	Spike/LFP	[33]
Thomas recording tetrodes	4	0–20 cm	Diameter: 95 μm	Spike/LFP	[34]
Thomas recording heptodes	7	0–20 cm	Diameter: 95 μm	Spike/LFP	[35]
Thomas recording 3D-heptodes	7	0–20 cm	Diameter: 95 μm	Spike/LFP	[36,37]
Thomas recording multitrodes	8, 16	0–20 cm	Diameter: 300 μm	Spike/LFP	[38,39]
Deep Array	32, 64, 128	90 mm	Diameter: 0.2 mm, site diameter: 20 μm	Spike/LFP	[44]
Microwire array	4–36	23 mm	Wiring diameter: 25, 50 μm	Spike/LFP	[47]
Microwire brush array	8–96	2.5–120 mm	Diameter: 12 μm	Spike/LFP	[48]
Utah electrode array	100	0.5–1.5 mm	Diameter: 80 μm	Spike/LFP	[53–55]
MERF	128	>1.9 mm	Diameter: 95 μm	Spike/LFP	[84]
Vector array	32	80 mm	Electrode width: 50–175 μm	Spike/LFP	[158] https://www.neuronexus.com/products/electrode-arrays/over-10-mm-depth/#ordering-information
Matrix Array	64, 128, 256	10 mm	*X*, *Y* , *Z* span: <1.8 mm, <3 mm, <15 mm	Spike/LFP	https://www.neuronexus.com/files/Matrix%20Array/MatrixArray-ConfigurationGuide.pdf
Neuroscroll probe	1,024	1–9 cm	Diameter: 84 μm, 138 μm	Spike/LFP	[86]
Neuralink	3,072	-	Thread length: 20 mm	Spike/LFP	[89,90]
DBS probe	4	>6.5 mm	Side length: 1.5 mm	Spike/LFP	[101]
Thin-film electrode	128	10 cm	Diameter: 0.8–1.27 mm	Spike/LFP	[104]
Carbon fiber	1,64	0.5–5 mm	Diameter: <10 μm	Spike/LFP	[110,127]

## Applications

In the application field, metal microelectrodes, silicon-based microelectrodes, flexible multi-channel electrodes, and deep brain electrodes related to major clinical brain diseases are mostly used for electrophysiological detection of the brain, while neurotransmitter chemical detection electrodes are widely used in neurochemical research [135,136]. Electrode array devices are widely used in neuroscience and BCI research fields, mainly for neuroscience research, neurological disease research, BCI technology, neuroplasticity research, neuropharmacology research, and so on.

### Implantation procedure

The selection of electrode materials for brain implantation is very important, and they must have good biocompatibility, including nontoxicity, biological stability, low sensitization, and anti-biological contamination [137–139]. In the long-term implantation process, the mechanical strength and biocompatibility of the electrode are very important, during which many changes may occur to the electrode, such as material elastic changes, molecular leaching, corrosion, and hydrolysis [140]. The outermost layer of the electrode implanted in the brain has high structural integrity, which can better avoid the release of debris caused by inflammatory reaction and electrode wear.

In the process of implantation, the electrode should be easy to implant without damaging the surrounding tissue and device itself, which has high requirements for material selection and mechanical properties. Rigid electrodes are easy to implant, but mechanical mismatch [141–144] can easily lead to stimulation and inflammatory response at the implantation site. The flexible electrode can reduce the mechanical stress and the abovementioned stimulate, but it needs a larger positioning device for electrode placement, increasing the complexity of the operation.

After electrode implantation, the brain may sustain damage during the procedure, potentially leading to inflammation, gliosis, and neuronal injury. Sorrentino et al. [145] collected neural tissue from DBS electrodes implanted in patients with Parkinson’s disease and essential tremor, enabling the detection of specific neuropathological markers, such as α-synuclein. This approach provides critical insights into the pathological characteristics of neurodegenerative diseases, contributing to a deeper understanding of disease mechanisms. Moreover, this detection method may facilitate the optimization of electrode materials, surface coatings, and implantation strategies, ultimately minimizing tissue damage and enhancing signal recording quality and long-term stability.

### Application of deep brain electrode array device in neuroscience research

One of the main application fields of primate deep brain electrode array is neuroscience research. By recording the discharge activities of single or multiple neurons, researchers can reveal the functions and regulatory mechanisms of neural circuits, and study the functions of brain regions, neural network activities, and cognitive processes. In primate research, researchers have developed various forms of electrode devices for single-cell discharge detection in the deep brain. The electrode arrays include metal microelectrodes, silicon-based microelectrodes, flexible multi-channel electrodes, and deep brain electrodes related to major clinical brain diseases. Each electrode can locate different deep brain nuclei, such as the striatum, hippocampus, and STN, to explore the functional role across various research domains.

Deep brain electrodes can also be applied in the study of spatial cognition, which can help reveal the neural mechanism of the brain in spatial perception and navigation by detecting electrophysiological or electrochemical signals in the organism. In addition, these electrodes are commonly used in visual research to help elucidate the neural mechanisms of visual information processing. Meanwhile, they can also be utilized to investigate synaptic connections and plasticity changes between neurons, providing insights into the mechanisms underlying learning, memory, and neural development, as well as the role of neural plasticity in these processes. This study is based on primates and laid a foundation for the detection of deep brain neural information in primates [146,147].

### Clinical application of deep brain electrode array device

Detecting deep brain neural information is of great significance for clinical application. Electrodes implanted in the brain contact with neurons to record electrical signals, which can be used to detect the brain activity in lesion location of subjects. Deep brain electrode array has very good clinical and scientific research value due to its high signal quality and good stimulation effect, which can lead to changes in neuronal firing mode [148]. The electrode arrays are crucial for researching DBS in the treatment of motor symptoms of patients with Parkinson’s disease [149] and the treatment of neurological diseases such as epilepsy. DBS is used to treat Parkinson’s disease by targeting specific brain regions, mainly STN and globus pallidum internus (GPi) [150]. By delivering high-frequency electrical impulses to these targets, the electrode can modulate pathological brain activity, which can restore normal motor function by reducing tremors and rigidity. This effect is believed to occur through the normalization of abnormal firing patterns in the basal ganglia circuitry, particularly by reducing overactivity in the STN and GPi. In epilepsy, the main target is the anterior thalamic nucleus (ATN), which is a key structure involved in the development of epileptic seizures [151]. Deep brain electrodes work by modulating the Papez circuits involved in seizure activity, potentially preventing the spread of abnormal electrical activity. The mechanism behind its effectiveness is related to the suppression of hyperexcitability in seizure-prone regions of the brain, stabilizing neuronal firing patterns and reducing the frequency of seizures. In this research, electrode array can record and regulate the discharge activity of relevant brain regions to achieve symptom relief. In clinical application, electrodes are usually used to detect the electrical activity or chemical reaction in organisms for diagnosis, monitoring, positioning, or treatment. The form and preparation process of electrodes in different application scenarios will vary according to specific application requirements. For example, the depth electrode of SEEG can help determine the location of the lesion and the origin of epilepsy, and DBS electrode is widely used to locate and regulate the neural activity of relevant brain regions in patients with Parkinson’s disease.

The BCI technology has been developed by coding the signals obtained by stimulating the spinal cord or brain, connecting the activities of brain neurons to external devices, and controlling external devices through the decoding of neural signals to improve the independence of patients with severe physical disability. Deep brain electrode array has a broad application prospect in BCI technology. It can record and decode brain activity and realize the development of human–computer interaction and brain control technology. Capturing electrophysiological signals and converting them into commands to control external devices, such as prosthetics and robots, can realize brain–computer interaction and motion recovery. The development of deep brain electrode array device provides a powerful tool for neuroscience and BCI research, which helps us better understand the function of the brain and the working principle of the nervous system.

Focused ultrasound (FUS) leverages acoustic waves to induce therapeutic mechanical or chemical changes, emerging as a novel approach in neuromodulation. Its noninvasiveness and high precision make it a potential alternative or complementary technique to DBS and traditional electrode technology [152]. Despite the fundamental differences between FUS and electrode stimulation and recording, potential integration strategies remain under exploration. First, FUS can temporarily open the blood–brain barrier, assisting the precise delivery of neurotransmitters or pharmacological agents, thereby enhancing the efficacy of electrode stimulation or neural signal recording. The combination of FUS and electrode multimodal neuromodulation approach may provide more precise targeted therapy, improve treatment efficacy, and reduce tissue reactions associated with long-term electrode implantation. However, further research is required to assess the impact of FUS on the surrounding neural tissue of implanted electrodes to ensure their compatibility.

The clinical use of invasive MEAs is an important step in the transformation of NHP brain electrodes. The safety, reliability, and clinical efficacy of electrodes are very critical [153], and it is particularly important to break through the limitations of these technologies.

## Summary and Outlook

In the past 20 years, substantial progress has been made in the detection and stimulation technology of deep brain neural information; especially, the deep brain electrode array device has emerged in neuroscience research and clinical application.

In terms of electrode performance, increasing the electrode surface area can reduce the resistance impedance and improve the signal-to-noise ratio. At the same time, in order to detect the signal of a single neuron and reduce tissue damage, it is also necessary to reduce the electrode surface area [154,155]. Therefore, the technology of surface modification of electrode sites has become the key to improve the performance of electrodes, such as coating conductive polymer materials on the electrode surface or modifying nanocomposites such as reduced graphene oxide nanoparticles [156], which help to reduce the impedance, improve the electrode performance, and increase the biocompatibility. Additionally, combined with MEMS, CMOS, laser cutting, 3D printing, and other technologies, increasing the number of channels of the electrode can improve the spatial resolution of recording neuronal signals. However, the development of biocompatible materials and microminiaturized high-precision manufacturing process are still challenging.

In terms of electrode functions, with the development of MEMS technology, the deep brain electrode can realize the detection of electrophysiological and electrochemical dual-mode signals. It helps to understand the complex functions and neural regulation mechanisms of the deep brain structure, and promote the progress of brain science research and the development of neurological disease treatment.

In terms of electrode application, although the deep brain electrode array device has been widely used in the treatment of severe diseases such as Parkinson’s disease and epilepsy, its potential has not yet been fully explored. The application of electrodes in the treatment of sleep disorders and spatial navigation cognitive research provides a new direction of exploration, which can help understand the brain’s cognitive and memory mechanisms. In general, the application of clinical electrodes and primate deep brain electrodes still needs further expansion.

Future research and technological innovation will further expand the understanding of deep brain neural activities and bring new opportunities and challenges for the further development of neuroscience. With the progress of technology, the deep brain electrode array device applied in primates will better meet the needs of scientific research and medical applications. It is expected that in the future, more innovative electrode array devices and analysis methods will be developed to promote the research of deep brain neural information to achieve greater breakthroughs.

## Data Availability

All data included in this study are available upon request by contact with the corresponding author.
